# E-health physical activity interventions and moderate-to-vigorous intensity physical activity levels among working-age women: a systematic review protocol

**DOI:** 10.1186/2046-4053-4-3

**Published:** 2015-01-14

**Authors:** Jennifer L Reed, Stephanie A Prince, Christie A Cole, Kara A Nerenberg, Swapnil Hiremath, Heather E Tulloch, J George Fodor, Agnieszka Szczotka, Lisa A McDonnell, Kerri-Anne Mullen, Andrew L Pipe, Robert D Reid

**Affiliations:** Division of Prevention and Rehabilitation, University of Ottawa Heart Institute, 40 Ruskin Street, Ottawa, ON K1Y 4W7 Canada; Division of General Medicine, Alberta Health Services, Calgary, AB T2N 1X9 Canada; Clinical Epidemiology Program, Ottawa Hospital Research Institute, Ottawa, ON K1Y 4E9 Canada; Berkman Library, University of Ottawa Heart Institute, 40 Ruskin Street, Ottawa, ON K1Y 4W7 Canada

**Keywords:** Motor activity, Women, e-health, Internet, Exercise, Movement, Chronic disease prevention

## Abstract

**Background:**

The rapid pace of modern life requires working-age women to juggle occupational, family, and social demands. Despite the large numbers of working-age women in developed countries and the proven benefits of regular moderate-to-vigorous intensity aerobic physical activity (MVPA) in chronic disease prevention, few women meet current physical activity (PA) recommendations of 150 min of MVPA per week. It is important that appropriate and effective behavioral interventions targeting PA are identified and developed to improve the MVPA levels of working-age women. As women worldwide embrace modern technologies, e-health innovations may provide opportune and convenient methods of implementing programs and strategies to target PA in an effort to improve MVPA levels and cardiometabolic health. Previous reviews on this topic have been limited; none have focused on working-age women from developed countries who exhibit inappropriately low PA levels. It remains unknown as to which e-health interventions are most effective at increasing MVPA levels in this population. The purpose of this systematic review is to examine the effectiveness of e-health interventions in raising MVPA levels among working-age women in developed countries and to examine the effectiveness of these interventions in improving the health of women.

**Methods:**

Eight electronic databases will be searched to identify all prospective cohort and experimental studies examining the impact of e-health interventions for increasing MVPA levels among working-age women (mean age 18–65 years) in developed countries. Gray literature including theses, dissertations, and government reports will also be examined. Study quality will be assessed using a modified Downs and Black checklist, and risk of bias will be assessed within and across all included studies using the Cochrane’s risk of bias tool and Grades of Recommendation, Assessment, Development and Evaluation approach. A quantitative synthesis in the form of meta-analyses for measures of MVPA and health outcomes will be conducted where possible.

**Discussion:**

This review will determine the effectiveness of e-health interventions in raising MVPA levels in working-age women in developed countries. It will form a contemporary, rigorously developed, and reliable research base for policy makers and stakeholders; and inform and influence the development and implementation of effective e-health interventions designed to increase MVPA levels and improve health outcomes in this population.

**Systematic review registration:**

PROSPERO CRD42014009534

## Background

Physical activity (PA) is a highly modifiable health behavior. Regular PA prevents or ameliorates several chronic conditions or health states, including, hypertension, dyslipidemia, overweight and obesity, cardiovascular disease, diabetes, anxiety, depression, certain cancers, and the risk of premature death [1–3]. World Health Organization (WHO) recommendations suggest that adults should accumulate at least 150 min of moderate-to-vigorous intensity aerobic PA (MVPA) each week [4]. Examples of MVPA include brisk walking, jogging, climbing, lifting heavier loads, swimming, and competitive sports. Despite the proven benefits of regular MVPA [1–3], few (3%–14%) working-age women in North America are meeting current MVPA recommendations [5, 6]. Not surprisingly, data from the recent Canadian Health Measures Survey (CHMS) and National Health and Nutrition Examination Survey (NHANES) revealed that 28%–31% of working-age women were classified as overweight, and 24%–36% were classified as obese [7, 8]. Further, an alarming proportion of working-age women in North America (2 of every 3 women) have established risk factors for cardiovascular diseases [9, 10], the leading cause of death in North America, including hypertension (estimates of 19%–32%) [11, 12], dyslipidemia (estimates of 11%–25%) [13, 14], and type II diabetes (estimates of 7%–11%) [15, 16].

The rapid pace of modern life requires working-age women to juggle occupational, family, and social demands. On most days of the week, working-age women in North America (47% of the total work force) spend a disproportionate number of their waking hours at work, while simultaneously contributing more to unpaid work (e.g. cooking, cleaning, child care, gardening) when compared to their male counterparts [17, 18]. Most of these unpaid household activities are not vigorous enough to meet the current MVPA recommendations [17, 19]. Women also lead the largest proportion (79%) of single-parent families [20] and earn 22%–33% less, on average, than males for equivalent full-time paid work [21–23]. Among these lower income families, the need to work overtime or acquire a second job is also common [24]. Many such women have little time to prioritize or optimize their health. Consequently, “lack of time” is one of the most commonly cited barriers to regular PA participation by working-age women [25].

It will be important that appropriate, effective e-health interventions targeting PA are identified, developed, and distributed to improve the MVPA levels of working-age women [5, 6]. E-health strategies offer a novel mode of delivery of PA interventions. North Americans are among the world’s heaviest internet users, and more than 95% of North American households have internet access [26, 27]. Women worldwide have embraced mobile technologies for communication, to receive information, and to access health care for themselves and their families [28]. Most (66%) new mobile subscribers are now women [26, 27]. As “smartphones” and mobile innovations have become more accessible and affordable, these technologies have assumed important new roles [28, 29]. E-health innovations may be very useful in influencing PA behavior, improving MVPA levels and subsequently impacting the cardiometabolic health of working-age women.

In general terms, e-health refers to the use of emerging information and communication technology, particularly the Internet, to improve or enable health and health care. The e-health approach encompasses a wide range of services or systems, including electronic health records, e-prescribing, telemedicine (e.g. online and telephone coaching), consumer health informatics (e.g. on-demand educational content), mobile devices (e.g. Fitbit, SenseWear, JawBone), and real-time monitoring of user health and behavioral data. These e-health activities, particularly telemedicine, consumer health informatics, and behavior monitoring, have significant potential to allow highly individualized behavior change interventions [30–33].

Previous reviews have reported beneficial effects of e-health interventions on several health parameters, including: PA levels (i.e. minutes/hours per week); fitness; nutritional practices; weight loss and maintenance; chronic disease management and medication adherence; psychosocial factors; work performance; and health risks and health-care cost outcomes among working-age adults [34–42]. There have been few evaluations, however, of e-health’s impact on MVPA levels [36, 37]; none have focused on working-age women or those from high-income Organization for Economic Co-operation and Development (OECD) countries [43] who typically exhibit poor adherence rates (≤50%) to current PA recommendations [6, 44]. The principal objective of this proposal is to systematically review the evidence of the effectiveness of e-health interventions in increasing MVPA levels in working-age women in developed countries. The secondary objective is to examine the effectiveness of e-health interventions in improving the known beneficial health sequelae of MVPA (e.g. improvements in weight, body mass index [BMI], body composition, waist circumference, blood pressure, lipid levels, glucose concentrations, quality of life, mental health).

## Methods

### Study design

A systematic review and meta-analysis will be undertaken to identify e-health interventions that have resulted in increased MVPA levels in working-age women in developed countries. The systematic review will adhere to the reporting guidelines of the “Preferred Reporting Items for Systematic Reviews and Meta-Analyses (PRISMA) statement” [45] and will meet the criteria outlined in “A Measurement Tool to Assess Systematic Reviews (AMSTAR) checklist” [46, 47].

### Study registration

This systematic review is registered with PROSPERO (registration number: CRD42014009534; http://www.crd.york.ac.uk/PROSPERO).

### Types of participants

Studies will be included if the sample is principally comprised of working-age women (≥80% women in the sample and/or where female data can be extracted) with a mean age between 18 and 65 years old living in high-income/developed countries (as per OECD criteria). Studies involving participants with a mean age lower than 18 years or greater than 65 years will be excluded.

### Types of exposures

Eligible studies must contain an intervention component delivered using an e-health process that is designed to increase MVPA levels. The interventions may include, but are not limited to: wearable technology (e.g. PA tracking devices); telemedicine (i.e. providing clinical care at a distance); mobile devices; and health informatics (i.e. electronic health resources). The delivery of the interventions may be single- or multi-modal.

### Types of comparators

Control groups will be used, when available, to compare effects. No restrictions will be placed on the control groups (e.g. no PA intervention, low intensity PA intervention, no e-health intervention, usual care).

### Types of outcomes

Eligible studies must report a measure of MVPA as the primary outcome for the systematic review and meta-analysis will be changed in minutes per day of MVPA. Of note, MVPA is defined as a behavior with an energy expenditure ≥3 metabolic equivalents (METs), ≥40% of VO_2_ reserve, ≥64% of peak heart rate, ≥12 rating of perceived exertion, or >100 steps per minute [19, 48–51]. Measures of time (e.g. minutes per day) spent engaging in MVPA and, where possible, a measure of variance around this outcome (e.g. standard error, 95% confidence intervals [CI]) will be extracted from all eligible and included studies regardless of the unit or method of MVPA measurement. MVPA can be either objectively measured (e.g. indirect calorimetry, accelerometers, pedometers, activity monitors) or self reported (e.g. questionnaire, journal, log). Further, MVPA can be described using a composite measure of total time spent in MVPA or separately for moderate and vigorous intensities. Secondary outcomes including potential and known beneficial health sequelae of MVPA (e.g. weight, BMI, body composition, waist circumference, blood pressure, lipid levels, glucose concentrations, quality of life, mental health) [1–3] will be extracted.

### Types of studies

We will include all experimental studies (RCTs, pre-post design, quasi-experimental) that examine the impact of e-health interventions on increasing MVPA levels among working-age women from developed countries. Only articles available in English or French will be included as the authors are proficient in these languages. If there is an adequate number of RCTs, a summary of the evidence and the confidence ascribed to it will be provided using the Grades of Recommendation, Assessment, Development and Evaluation (GRADE) approach [52] to increase the internal validity of the review. RCTs receive the highest grade with this approach.

### Search strategy methods for the identification of primary studies

A comprehensive and sensitive search strategy was designed in collaboration with a research librarian (EW) and was peer reviewed by a second research librarian (AS). The search strategy includes a search of eight electronic databases: Ovid MEDLINE(R) In Process & Other Non-Indexed Citations (1946 to present); EBM Reviews—Cochrane Database of Systematic Reviews (2005 to present); EBM Reviews—Cochrane Central Register of Controlled Trials (1991 to present); EMBASE Classic + (1947 to present); CINAHL (1981 to present); Ovid PsycINFO (1806 to present); SPORTDiscus (1949 to present); and, Dissertations and Theses (1980 to present). The strategy is illustrated using the Medline search as an example (Table 1) and will be modified according to the indexing systems of the other databases. Gray literature (non-peer reviewed works) that meets the inclusion criteria will be obtained including published lists of theses and dissertations, government reports, and unpublished data and manuscripts (provided by original authors). Government reports will be searched using the Google search engine and a combination of key text words. Unpublished data and manuscripts will be solicited from original authors of studies that report on collecting MVPA. The bibliographies of all investigations selected for the review, as well as those of previous reviews, will be examined to identify further studies. The Google search engine will be used to identify studies published in non-indexed journals.

**Table 1:** **Sample Medline search strategy**

1	(eHealth or e-Health).tw. (1900)
2	Cellular Phone/(4894)
3	Text Messaging/(675)`
4	Internet/ (52190)
5	((cell* or mobile or smart) adj phone*).tw. (5330)
6	text messag*.tw. (1198)
7	(smartphone* or iPhone*).tw. (1420)
8	((mobile or phone or iPhone or smartphone) adj2 (app or apps or application*)).tw. (1096)
9	internet.tw. (30384)
10	web-based.tw. (15811)
11	Computer-Assisted Instruction/(9671)
12	computer-tailored.tw. (223)
13	(mobile adj (health or tech* or device* or telephone*)).tw. (2114)
14	(exergam* or exer-gam*).tw. (112)
15	Wii.tw. (427)
16	((fitness or activ* or exercis* or interactive) adj2 (videogam* or “video gam*” or gaming)).tw. (241)
17	Accelerometry/(1004)
18	acceleromet*.tw. (7922)
19	pedomet*.tw. (1671)
20	Electronic Mail/(1953)
21	(email* or e-mail* or “electronic mail*”).tw. (13868)
22	((wearable* or wear-able*) adj3 (monitor* or sensor* or sensing or device* or tech*)).tw. (1434)
23	(activity adj (monitor or monitors or sensor*)).tw. (1458)
24	(motion adj (sensor* or monitor or monitors)).tw. (549)
25	fitbit.tw. (10)
26	activPal*.tw. (112)
27	MyFitnessPal.tw. (1)
28	NikeFuel.tw. (1)
29	Sensewear.tw. (196)
30	Omron.tw. (419)
31	m-health.tw. (120)
32	personal digital assistant*.tw. (941)
33	pda.tw. (6319)
34	Computers, Handheld/(2495)
35	telemedicine/(12203)
36	or/1-35 (132265)
37	Motor Activity/(83314)
38	exp Exercise/(126776)
39	Physical Fitness/(22843)
40	“Physical Education and Training”/(12094)
41	exp Exercise Therapy/(32132)
42	Movement/(62312)
43	Bicycling/(8022)
44	(physical* adj (activit* or exercise* or fitness)).tw. (77935)
45	((fitness or exercise) adj (class* or course* or program* or training)).tw. (18953)
46	(“aerobic exercis*” or aerobics).tw. (5957)
47	(walk* or run* or bike or bicycl*).tw. (225182)
48	yoga.tw. (2202)
49	Yoga/(1637)
50	((moderate or high or vigorous) adj “intensity activit*”).tw. (496)
51	(moderate-vigorous adj2 activit*).tw. (196)
52	(“moderate to vigorous” adj2 activit*).tw. (2113)
53	mvpa.tw. (1484)
54	exercise.ti. (79752)
55	or/37-54 (550499)
56	Intervention Studies/(7425)
57	intervention*.tw. (597146)
58	Program Evaluation/(47941)
59	evaluation studies/(204980)
60	Multicenter Study/(187025)
61	Observational Study/(5743)
62	(observational adj (study or studies)).tw. (50399)
63	Randomized Controlled Trials as Topic/(99016)
64	randomized controlled trial/(397704)
65	randomi?ed.tw. (405610)
66	exp Clinical Trials as Topic/(293501)
67	clinical trial/(500151)
68	controlled clinical trial/(90513)
69	(clinical adj trial*).tw. (233882)
70	case–control studies/(196739)
71	exp Cohort Studies/(1426840)
72	Meta-Analysis/(53863)
73	(meta-analysis or metaanalysis).tw. (60993)
74	“review”/(1969675)
75	(systematic adj (review$1 or overview$1)).tw. (61023)
76	or/56-75 (4795829)
77	36 and 55 and 76 (5733)

### Selection of eligible studies

Articles will be imported into Microsoft Excel (Microsoft Canada Inc. Mississauga, ON, Canada), and all duplicates will be removed; only the most relevant article per data source/analysis will be retained. Two independent reviewers (JLR, CAC) will screen the titles and abstracts of all articles to identify potentially relevant articles. Full texts of each potentially relevant article identified by either reviewer during the title and abstract screening phase will be reviewed to determine whether the title and abstract screening inclusion criteria are met. The full texts of all potential articles that meet the inclusion criteria will be obtained and reviewed. Two independent reviewers will screen the full texts for inclusion (JLR, CAC). Any disagreements between the reviewers will be resolved by consensus and/or discussion with a third reviewer (SAP). Intra-class correlations will be calculated to assess agreement between the reviewers. Reviewers will not be blinded to the authors or journals when screening articles.

### Data extraction

A data extraction form will be created, prior to data extraction, using a subset of the included studies. The extraction form will be modified following feedback from the research team to improve its usability and ensure that complete and pertinent data is obtained. Standardized data abstraction forms, including quality assessments, will be completed by both reviewers (JLR and CAC). Any disagreements will be resolved by consensus and/or discussion with a third reviewer (SAP or RDR). Reviewers will not be blinded to the authors or journals when extracting data.

From each study, the following data will be extracted: publication details (authors, year, country of study); participant characteristics (age range, mean age, sex distribution, chronic diseases, health states, population, setting); sample size; study design (RCT, pre-post, quasi-experimental); time points when data were collected (e.g. 3 weeks, 4 months); length of follow-up; intervention details; description of control group; information regarding study methods (e.g. blinding and randomization techniques); MVPA measurement method and whether self-report or objective tools were used; MVPA units of measurement; statistical analyses methods (i.e. *t*-tests, linear modeling); effect of the intervention on MVPA (effect size, 95% CI, standard mean error or deviation); and effect of intervention on known beneficial health sequelae of MVPA (weight, BMI, body composition, waist circumference, blood pressure, lipid levels, glucose concentrations, quality of life, mental health) [1–3]. In cases where several publications report the same results from the same data source, only one article per data source/analysis will be retained to avoid double counting. When an investigator uses a measure of MVPA (e.g. FITT log, accelerometers) but does not report on these outcomes in the manuscript, or if a paper reports only a study protocol, the authors will be contacted to determine whether MVPA results can be obtained; other missing data to determine inclusion criteria (e.g. study design, age distribution, sex distribution) will also be obtained. A maximum of two e-mail or phone call attempts will be made to contact the corresponding authors of these articles to obtain additional data.

### Quality assessment and risk of bias within studies

The Downs and Black checklist will be used to assess the quality and risk of bias of the primary studies [53]. This checklist contains 27 items, with a maximum possible score of 32 points [53]. We will simplify the scoring of item 27 from a five-point range to a binary system, granting one point (1) for adequate power calculations or no points (0) if power was not adequately addressed. The maximum possible score for this modified checklist will be 28 points with higher scores indicating superior quality. The quality of the individual studies will be rated by reviewer CAC and verified by reviewer JLR. The quality scores will be used for performing subgroup analyses (high-quality vs. low-quality). The Cochrane Collaboration’s tool will be used to assess risk of bias for each RCT. Items included in Cochrane’s risk of bias assessment include: sequence generation (randomization); allocation concealment; blinding of participants, personnel and investigator; incomplete data (e.g. losses to follow-up, intention-to-treat analysis); selective outcome reporting; and other possible sources of bias. The risk of bias assessment will be carried out by two independent assessors (JLR and CAC); any disagreements between assessors will be resolved by consensus and/or through discussion with a third reviewer (SAP).

### Overall quality of the evidence

The quality of the evidence for the RCTs will be assessed as high, moderate, low, or very low using the Grading of Recommendations Assessment, Development and Evaluation (GRADE) approach. With the GRADE approach, the highest quality rating is for RCT evidence. In addition to study design, the quality of evidence will be rated upon possible risk of bias, imprecision, heterogeneity, indirectness, or suspicion of publication bias. Risk of bias for the RCTs will be assessed using Review Manager (RevMan) 5.3.3 (The Nordic Cochrane Centre, The Cochrane Collaboration, 2012) [54], the Cochrane Collaboration’s software to prepare and maintain systematic reviews, and then imported into GRADEprofiler (GRADEpro) Version 3.6.1 [55] to create a summary of findings table and rate the quality of the evidence using the GRADE approach.

### Planned analyses

We will perform a quantitative synthesis and present results in the form of forest plots and meta-analyses only when the included studies are sufficiently homogenous in terms of study design, participants, interventions, and outcomes to provide meaningful summary measures. The forest plots and meta-analyses will be created using RevMan 5.3.3 to synthesize the measures of effect (e.g. mean differences) and 95% CI for each intervention on minutes per day spent engaging in MVPA from baseline. A random-effects model will be used as effect sizes are likely to be similar but not identical across all studies. Statistical heterogeneity will be assessed using the I^2^ statistic with values above 75% and *p* < 0.10 used to indicate high heterogeneity across studies [56]. If high heterogeneity is found, a meta-analysis will not be performed. A funnel plot of the included studies’ estimates of effect sizes will be used to assess the presence of publication bias. Funnel plots will only be performed if 10 or more studies are included. The plots will be assessed both visually and by using Egger’s test, with *p* < 0.10 used to indicate the presence of a significant publication bias [57].

### Subgroup analyses

Several subgroup analyses will be performed if sufficient data are available. These analyses will examine differences between: age (e.g. 18–24 years vs. 25–44 years vs. 45–65 years); education (e.g. high school vs. post-secondary vs. graduate); number of children; marital status (married vs. unmarried); working status (casual vs. full-time vs. part-time); country; income; self-reported and objectively measured MVPA; intervention focus (e.g. text messaging vs. online web forums vs. virtual care programs vs. feedback from wearable PA devices vs. mobile smart phone application vs. telephone); intervention mode (e.g. wearables vs. telemedicine vs. mobile devices vs. health informatics vs. multi-component vs. single-component); study design (e.g. control vs. no control group, randomized vs. non-randomized controlled trial); and impact on known beneficial health sequelae of MVPA (e.g. weight vs. BMI vs. body composition vs. waist circumference vs. blood pressure vs. lipid levels vs. glucose concentrations vs. quality of life vs. mental health). Of these subgroup analyses, intervention focus and mode may be the most important as it remains unknown which e-health interventions are most effective at increasing MVPA levels in working-age women in developed countries.

## Discussion

This systematic review will be the first, to our knowledge, to determine the effectiveness of e-health interventions in increasing MVPA levels among working-aged women in developed countries. The findings from this review will provide a contemporary, rigorous, and reliable research base to support policy makers and other stakeholders as they design and implement effective e-health interventions to address MVPA levels among working-age women in developed countries. The findings from this review will be disseminated through scientific peer-reviewed publications, conference presentations, and proceedings. The review authors will disseminate the findings to health researchers and academic institutions through national and international seminars and workshops.

## Abbreviations

MVPA: moderate-to-vigorous intensity physical activity
PA: physical activity
WHO: World Health Organization
CHMS: Canadian Health Measures Survey
NHANES: National Health and Nutrition Examination Survey
BMI: body mass index
OECD: Organization for Economic Co-operation and Development
PRISMA: Preferred Reporting Items for Systematic Reviews and Meta-Analyses
AMSTAR: A Measurement Tool to Assess Systematic Reviews
RCT: randomized controlled trials
METS: metabolic equivalents
GRADE: Grades of Recommendation Assessment, Development and Evaluation.
